# Tripartite phase separation of two signal effectors with vesicles priming B cell responsiveness

**DOI:** 10.1038/s41467-020-14544-1

**Published:** 2020-02-12

**Authors:** Leo E. Wong, Arshiya Bhatt, Philipp S. Erdmann, Zhen Hou, Joachim Maier, Sona Pirkuliyeva, Michael Engelke, Stefan Becker, Jürgen Plitzko, Jürgen Wienands, Christian Griesinger

**Affiliations:** 1 Department for NMR-based Structural Biology, Max Planck Institute for Biophysical Chemistry, Am Faßberg 11, 37077 Göttingen, Germany; 2 Institute of Cellular and Molecular Immunology, Georg August University of Göttingen, Humboldtallee 34, 37073 Göttingen, Germany; 3 Department of Molecular Structural Biology, Max Planck Institute of Biochemistry, Am Klopferspitz 18, 82152 Martinsried, Germany

**Keywords:** Cryoelectron tomography, Intrinsically disordered proteins, Signal transduction, Solution-state NMR

## Abstract

Antibody-mediated immune responses rely on antigen recognition by the B cell antigen receptor (BCR) and the proper engagement of its intracellular signal effector proteins. Src homology (SH) 2 domain-containing leukocyte protein of 65 kDa (SLP65) is the key scaffold protein mediating BCR signaling. In resting B cells, SLP65 colocalizes with Cbl-interacting protein of 85 kDa (CIN85) in cytoplasmic granules whose formation is not fully understood. Here we show that effective B cell activation requires tripartite phase separation of SLP65, CIN85, and lipid vesicles into droplets via vesicle binding of SLP65 and promiscuous interactions between nine SH3 domains of the trimeric CIN85 and the proline-rich motifs (PRMs) of SLP65. Vesicles are clustered and the dynamical structure of SLP65 persists in the droplet phase in vitro. Our results demonstrate that phase separation driven by concerted transient interactions between scaffold proteins and vesicles is a cellular mechanism to concentrate and organize signal transducers.

## Abstract (web summary)

Antibody-mediated immune responses rely on antigen recognition by the B cell antigen receptor (BCR) and SLP65 is a key scaffold protein mediating BCR signaling. Here authors show that effective B cell activation requires tripartite phase separation of SLP65, CIN85, and lipid vesicles.

## Introduction

Ligation of the BCR by cognate antigen results in the recruitment and activation of the cytosolic spleen tyrosine kinase Syk that in turn phosphorylates the enzymatically inert adapter protein SLP65^1^, also called B cell linker protein (BLNK)^2^. Apart from its C-terminal SH2 domain, SLP65 is an intrinsically disordered protein (IDP)^3^ that contains multiple tyrosine phosphorylation sites and proline-rich motifs (PRMs) to interact in a specific manner with SH2 and SH3 domain-containing ligands, respectively^4,5^. Phosphorylated SLP65 recruits Bruton’s tyrosine kinase (BTK) and Phospholipase C-γ2 (PLCG2)^6–8^ to trigger several intracellular B cell activation pathways such as mobilization of the second messenger Ca^2+^ and nuclear translocation of the pivotal transcription factor NF-κB^9,10^. Hence, SLP65 is the signal gatekeeper of B cell activation. Indeed, loss of SLP65 expression or its function severely compromises the development and function of B cells in mice and humans^11,12^.

More recently, we found that the signaling function of SLP65 relies on its interaction with intracellular vesicles to which it is attached by virtue of an N-terminal lipid binding moiety^13,14^. A majority of these vesicles stained positive for vesicle-associated membrane protein (VAMP) 7 and quinacrine, but there was no unique marker or combination of markers characterizing a particular cellular vesicle pool that specifically attracts SLP65 molecules. However, the vesicular compartmentalization of SLP65 appeared to be critical for the stimulation-independent (i.e. constitutive) association of SLP65 with CIN85^13,15^. The association of SLP65 and CIN85 is caused by atypical SH3 domain docking motifs in SLP65 that associate in a promiscuous manner with the three N-terminal SH3 domains of CIN85^15^. A C-terminal coiled coil domain of CIN85 forms stable CIN85 trimers that drive multimerization of SLP65. Functional studies showed that the formation of a permanent SLP65/CIN85 oligomeric complex in resting B cells is mandatory for proper B cell activation^4,16^. In humans, the loss of CIN85 expression causes severe antibody deficiency caused by B cell-intrinsic signaling defects, highlighting the non-redundant function of CIN85 for BCR signal transduction^17^.

To elucidate how exactly the concerted interactions between SLP65, CIN85 and vesicles promote B cell activation, we used a combination of various methods to investigate the structure of these complexes and their functional signaling role in the live B cell. Here we show that all three components act in concert to drive a tripartite liquid-liquid phase separation process in vitro at physiological protein concentrations. In the live B cell, this results in the formation of intracellular signaling bodies or droplets that are reminiscent of other phase-separated organelles such as stress granules or RNA transport granules^18–20^.

## Results

### Tripartite phase separation at physiological concentrations

**Fig. 1:**
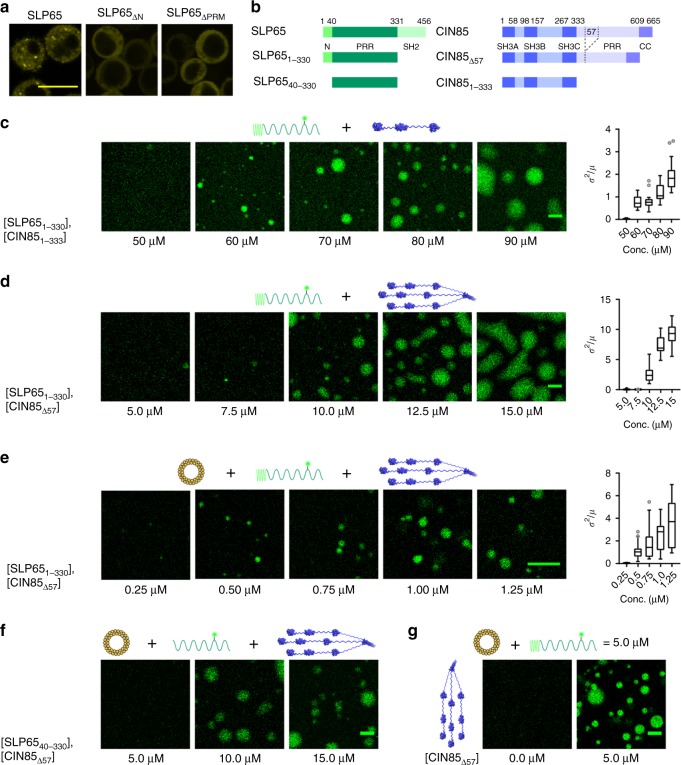
Tripartite phase separation of SLP65 with CIN85 and SUVs. **a** Confocal fluorescence microscopy of SLP65-deficient DT40 B cells that had been reconstituted with a citrine fusion protein of either wild-type SLP65 (left), a variant lacking N-terminal 45 amino acids (ΔN, middle), or a CIN85 binding-deficient variant (ΔPRM, right). **b** Domain architecture of the different constructs of SLP65 and CIN85 studied here (PRR: proline-rich region, CC: coiled coil). **c**–**e** Confocal fluorescence microscopy of a mixture of different concentrations of Atto 430LS-tagged SLP65_1-330_ with the corresponding equal concentrations of either (**c**) CIN85_1-333_, (**d**) CIN85_Δ57_, or (**e**) CIN85_Δ57_ and SUVs made from 1 mM of phospholipids (see Method). The SUVs had a lipid composition of DOPC:DOPE:DOPS = 65:25:10 mol% and a mean hydrodynamic radius of 23 nm. Concentrations of CIN85_Δ57_ refer to the monomeric concentration. Index of dispersion of the fluorescence intensity were shown as box-and-whisker plots (12 ROIs and two independent samples, *n* = 24). The line inside the box and the edges of the box correspond to the median, first and third quartiles, respectively. The outliers with value ≥1.5 times the interquartile range away from the top or bottom of the box are denoted by circles. Source data are provided as a Source Data file. **f** Different concentrations of Atto 430LS-tagged SLP65_40-330_ were mixed with the corresponding equal concentrations of CIN85_Δ57_ together with 1 mM of SUVs. **g** Mixture of 5 μM of Atto 430LS-tagged SLP65_1-330_ and 1 mM of SUVs without CIN85 (left) or with 5 μM of CIN85_Δ57_ (right). Scale bars equal to 10 μm (**a**, **c**–**g**).

In accordance with previously published data^13,15^, citrine-tagged wild-type SLP65 but not mutant versions of SLP65 lacking either the N-terminal lipid binding domain or the CIN85-interacting PRMs formed submicrometer granules in resting B cells (Fig. 1a). The subcellular localization patterns strictly correlated with the ability or disability of SLP65 to trigger mobilization of the second messenger Ca^2+^ on BCR ligation (Supplementary Fig. 1). Hence, the formation of granules through coordinated interactions of SLP65 with both vesicles and CIN85 appeared requisite for proper SLP65 signaling as suggested previously^13,15^. Next, we investigated by confocal fluorescence microscopy, whether granule formation can be recapitulated in vitro using recombinantly expressed SLP65 and CIN85 and synthetic lipid vesicles in different combinations. We found that in the absence of vesicles, SLP65 undergoes phase separation when mixed with a monomeric CIN85_1-333_ version lacking the trimerizing C-terminal coiled coil domain at and above 60 µM concentration of both proteins (Fig. 1b–c). The threshold concentration for phase separation was lowered to 10 µM when SLP65 was mixed with trimeric CIN85_Δ57_ (Fig. 1d). This CIN85 variant lacking 57 amino acids in the center of the protein was used because full-length CIN85 could not be recombinantly produced in sufficient quantities. However, we confirmed the functionality of the CIN85_Δ57_ version by reconstitution experiments of CIN85-deficient B cells (Supplementary Fig. 2). These results showed that SLP65 and CIN85 can undergo phase separation and that the phase separation boundary is controlled by their valency, which was mediated by three and nine SH3 domains of the monomeric and trimeric CIN85 forms, respectively. The promoting role of high valency for droplet formation is a common feature of phase-separating proteins^21^.

The SLP65/CIN85 droplets formed at and above 10 µM were about 10 µm in diameter, which is similar to or even larger than the diameter of the B cell itself. We therefore tested a possible contribution of vesicles and investigated whether the presence of small unilamellar vesicles (SUVs) in our in vitro approach would change the diameter of the droplets and/or lower the protein concentration needed for initiating the phase separation process. Indeed, addition of SUVs promoted droplet formation at 20 times lower protein concentrations, namely 0.5 μM (Fig. 1e). Importantly, this concentration was similar to the endogenous concentrations of SLP65 and CIN85 in DG75 and primary human B cells (Supplementary Fig. 3). Thus, phase separation of SLP65 and CIN85 occurs in vitro at protein concentrations found in vivo, but only in the presence of vesicles. This finding was confirmed further by the increased phase transition threshold of 10 µM for the N-terminal SLP65 deletion mutant, SLP65_ΔN_ (Fig. 1f), which is unable to attach to vesicles^13^ and that does neither support granule formation nor BCR-activated Ca^2+^ mobilization when expressed in live B cells (see Fig. 1a and Supplementary Fig. 1). Importantly, no phase separation was observed when SLP65 was mixed with SUVs in the absence of CIN85_Δ57_ (Fig. 1g) demonstrating again the concerted action of all three components that is needed for droplet formation in vitro. This finding recapitulated in vivo requirements of droplet formation and signaling. When SLP65-deficient B cells were reconstituted with a SLP65 variant that contained Arg-to-Ala substitutions in two of its PRMs to prevent binding to CIN85, granules were absent and the Ca^2+^ flux response was abrogated (Fig. 1a and Supplementary Fig. 1). Finally, the in vitro-assembled droplets were 1–2 µm in diameter, which closely resembled that of the granules observed in live B cells (Fig. 1a, e). Taken together, these results demonstrated that all three components necessary for in vitro phase separation are also essential for the proper subcellular localization and signaling competence of SLP65 in vivo. The vesicular attachment of SLP65 appears to provide a local seeding point that concentrates SLP65 to facilitate the recruitment of CIN85 and to initiate phase separation.

### SUVs rather than LUVs drive phase separation

**Fig. 2:**
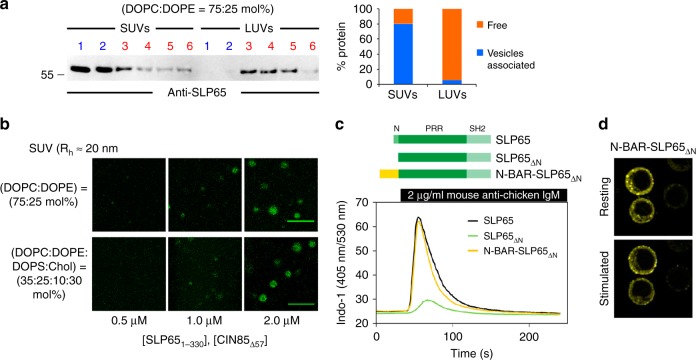
Phase separation modulated by vesicle curvature and lipid composition. **a** A mixture of recombinant SLP65 with either SUVs (Rh ≈ 20 nm) or LUVs (Rh ≈ 60 nm) was fractionated by a sucrose gradient flotation assay, and the different layers were subjected to anti-SLP65 immunoblot analysis (apparent MW indicated in kDa). Vesicles will float to the top and are found in layers 1 and 2. Bar graph shows the percentage of SLP65 associated with vesicles based on the immunoblot signal intensity. **b** Confocal fluorescence microscopy of a mixture of different concentrations of Atto 430LS-tagged SLP65_1-330_ and CIN85_Δ57_ together with SUVs containing DOPC:DOPE of 75:25 mol% (**b**, top) and DOPC:DOPE:DOPS:cholesterol of 35:25:10:30 mol% (**b**, bottom), respectively. (Scale bar = 10 μm) Source data are provided as a Source Data file. **c** SLP65-deficient DT40 B cells were transduced with constructs encoding citrine-tagged SLP65_ΔN_ fusion proteins with the N-BAR domain of Amphiphysin. Wild-type SLP65 and SLP65_ΔN_-expressing cells served as positive and negative controls, respectively. The domain structures of the expressed SLP65 variants are depicted in the upper part of (**c**). Cells were loaded with the Ca^2+^-sensitive fluorophore Indo-1 and Ca^2+^ flux was monitored by flow cytometry. Cells were stimulated with anti-BCR antibody (M4) at the indicated time point. **d** Confocal fluorescence microscopy of cells transduced with N-BAR-SLP65_ΔN_ were either left untreated (upper panel) or stimulated via their BCR (lower panel).

To further elucidate the role of vesicles in promoting droplet formation of SLP65/CIN85 complexes, we first tested the impact of the vesicle radius. We generated two types of neutral liposomes composed of 75 mol% 1,2-dioleoyl-sn-glycero-3-phosphocholine (DOPC) and 25 mol% 1,2-dioleoyl-sn-glycero-3-phosphoethanolamine (DOPE) with mean hydrodynamic radii (*R*_h_) of 20 nm and 60 nm, representing SUVs and large unilamellar vesicles (LUVs), respectively. Liposomes were mixed with recombinantly produced wild-type SLP65 and subjected to gradient ultracentrifugation upon which vesicles floated on the top of the tube. Individual fractions were tested for the presence of SLP65 by immunoblotting. This type of liposome floatation assay revealed a striking binding preference of SLP65 for SUVs with *R*_h_ of 20 nm (Fig. 2a). Furthermore, these neutral SUVs promoted phase transition of SLP65 and CIN85 with a transition threshold of 1 µM that was similar to that for negatively charged SUVs containing 30 mol% of cholesterol (Fig. 2b) – a common lipid composition reported for vesicles in mammalian cells^22^.

These data showed that the tripartite phase transition is facilitated by SUVs rather than LUVs, indicating that SLP65 attaches preferentially at small vesicles. Hence, the N-terminal lipid binding domain of SLP65 appeared to be sensitive to membrane curvature. To further investigate whether other SUV binding domains covalently connected to SLP65 would show phase separation and be functional in B cells, we exchanged the wild-type SLP65 N-terminus for another curvature sensing domain, the N-BAR (Bin-Amphiphysin-Rvs) domain of Amphiphysin, and expressed the resulting chimeric fusion protein in SLP65-negative DT40 B cells in order to then test its subcellular localization and signaling capacity (Fig. 2c–d). The N-BAR domain was reported to have a pronounced binding preference for small, i.e. highly curved, vesicles^23,24^. The chimeric N-BAR-SLP65_ΔN_ was expressed in SLP65-deficient B cells in similar amounts and fully restored Ca^2+^ mobilization to wild-type level (Fig. 2c). The signaling-competent N-BAR-SLP65_ΔN_ protein also formed dot-like speckles (Fig. 2d) that were reminiscent of those observed for wild-type SLP65 granules (see above). Furthermore, N-BAR-SLP65_ΔN_ translocated to sites of BCR activation at the plasma membrane following BCR ligation (Fig. 2d). Collectively, these data showed that the N-terminal lipid binding domain preferentially attaches at small vesicles with curved membranes and that this feature allows for tripartite phase separation at physiological concentrations, i.e. in vitro as well as in vivo.

### Vesicle clustering in the droplet phase revealed by cryo-ET

**Fig. 3:**
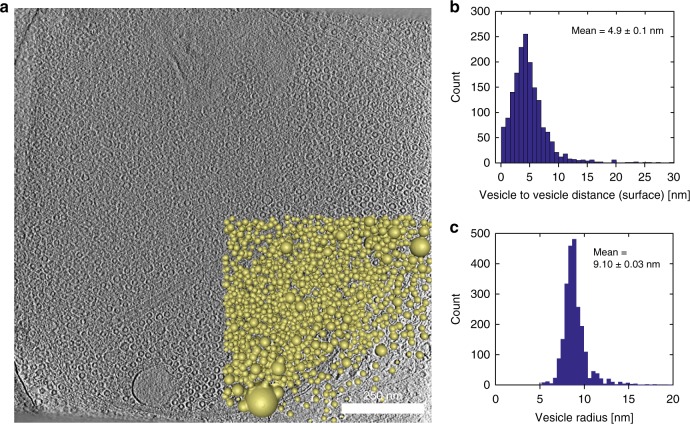
Cryo-electron tomogram of the tripartite droplets. **a** A mixture of 1 μM of SLP65_1-330_ and 1 μM of CIN85_Δ57_ together with 1 mM of SUVs was placed on a carbon grid and plunged frozen after incubation. The droplet shows a clear increase in concentration of vesicles toward its center with a steep fall-off at the edges. Golden spheres represent vesicles that were manually segmented for 3D-visualization in AMIRA 6.2 and processed using an in-house python script and Matlab. **b** Histogram of distances between vesicles with an average vesicle to vesicle distance of 4.92 ± 0.1 nm (mean ± SEM; *n* = 2349). **c** Histogram of the vesicle’s radii with a mean radius of 9.10 ± 0.03 nm (mean ± SEM; *n* = 1718). Source data are provided as a Source Data file.

Since SUVs are required for SLP65 and CIN85_Δ57_ to phase-separate at their physiological concentrations, we sought to investigate the ultrastructural organization within the droplets by cryo-electron tomography (cryo-ET) and assess their distribution in the in vitro phase-separated samples (Fig. 3a). The cryo-ET data showed that while the center is tightly packed, the vesicle concentration drops quickly toward the phase border. Furthermore, the tomograms showed a stochastic distribution of SUVs inside the droplets and an average surface-to-surface distance of 5 nm (Fig. 3b). The radius of vesicles within the phase-separated compartment was found to be 9.10 ± 0.03 nm (Fig. 3c). Taken together, the tomography data therefore supports the idea that formation of the droplets is driven by tripartite phase separation between SLP65, CIN85, and highly curved vesicles.

### SLP65 is 10-fold enriched in the droplet phase

We also characterized phase-separated SLP65/CIN85 droplets and assessed how strongly SLP65 is enriched in the droplet phase by quantification of its partition coefficients using the fluorescence intensity (Supplementary Fig. 4). This approach revealed that in the absence of SUVs, concentration of SLP65 in the droplet phase was found to be three to ten times higher than in the dispersed phase. Despite the moderate difference in concentration, distinct spherical liquid phases were also observable by bright-field imaging (Supplementary Fig. 5). Importantly, the addition of SUVs resulted in an augmented ten-fold enrichment of SLP65 when mixed at the transition threshold concentration of 0.5 μM, and the partition coefficient further increased with higher concentrations of SLP65 and CIN85_Δ57_ (Supplementary Fig. 4). These data further emphasize the importance of SUVs in the formation of phase-separated SLP65/CIN85 signaling complexes.

### Phase-separated SLP65 retains dynamic flexibility

**Fig. 4:**
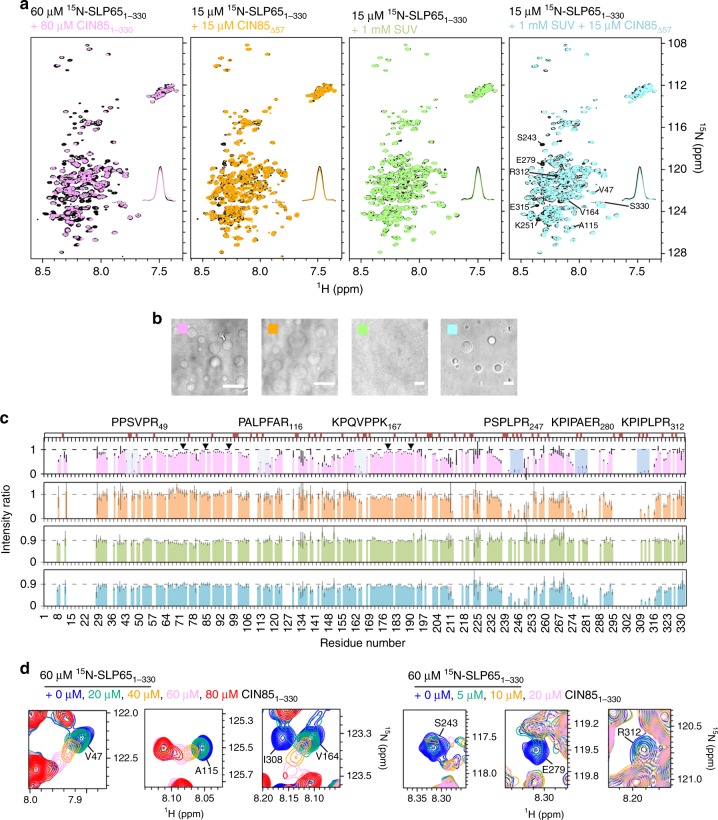
Structural properties of phase-separated SLP65 and its interactions with CIN85. **a** ^15^N-HSQC spectra of 60 µM or 15 µM of ^15^N-labeled SLP65 only (black) were overlaid by the spectra of SLP65 after addition of unlabeled CIN85_1-333_ (pink), CIN85_Δ57_ (orange), SUVs (green), or SUVs and CIN85_Δ57_ (cyan) at the indicated concentrations. **b** Bright field images of the respective NMR samples denoted by colored boxes. (Scale bar = 10 µm) **c** Residue-specific peak intensity ratio of the ^15^N-HSQC spectra shown in **a** of SLP65 with CIN85_1-333_ (pink), CIN85_Δ57_ (orange), SUVs (green), or SUVs and CIN85_Δ57_ (cyan) over SLP65 only at the corresponding concentrations. Error bar indicates uncertainty propagated from the peak’s signal-to-noise. Proline residues were marked in red on top of the chart, while PRMs that interact with CIN85 were highlighted with their sequences written. The five tyrosine residues that are phosphorylated by Syk (Y72, Y84, Y96, Y178, and Y189) were pointed out by inverted triangle. Source data are provided as a Source Data file. **d** Zoomed-in regions of ^15^N-HSQC spectra of 60 µM SLP65 titrated with increasing amounts of CIN85_1-333_ were overlaid to show either shifting or disappearance of the respective peaks associated with different PRMs.

Finally, we also characterized phase-separated SLP65 by NMR spectroscopy to gain structural insight at the atomic level. The sharp peaks observed in the ^15^N-HSQC (heteronuclear single quantum coherence) spectrum of SLP65 are typical for IDPs, implying fast reorientation motion of protein segments. Compared to SLP65 alone, phase-separated SLP65 induced by the addition of either CIN85_1-333_, CIN85_Δ57_, or CIN85_Δ57_ and SUVs exhibited similarly sharp peaks, as exemplified by the almost equal peak intensity for residues away from the binding sites (Fig. 4a–c) as well as the ^15^N relaxation data (Supplementary Fig. 6). These results showed that fluidity is completely preserved in phase-separated SLP65 in the absence, as well as in the presence of vesicles. Hence, functionally relevant motifs within SLP65, e.g. tyrosine phosphorylation sites, are equally well accessible under both conditions. Indeed, Syk-phosphorylated SLP65 retains its potential to phase-separate with CIN85 and SUVs. (Supplementary Fig. 7). Accordingly, the five tyrosine phosphorylation sites are not involved in the binding to CIN85’s SH3 domains and therefore not expected to interfere with the SLP65/CIN85 interaction.

For the mixtures containing SUVs, the observed 10% signal loss in the NMR spectra (Fig. 4c, depicted in green and cyan) was due to fractions of SLP65 interacting with SUVs that rotate much slower and did not depend on phase separation, i.e. on the presence or absence of CIN85_Δ57_. Backbone dynamics of SLP65 was not affected significantly by phase separation due to the relatively low partition coefficients (Supplementary Fig. 4). When concentrations of SLP65 and CIN85_1-333_ were increased from 120 μM to 2400 μM, the ^15^N lines were broadened by 1.7-fold only (Supplementary Fig. 6), indicating a modest increase in viscosity upon a substantial increase in protein concentration. This is in line with the NMR studies on phase-separated FUS LC^25^ and Ddx4^26^ that revealed fluidity reduction by a factor of 4–6 when concentrations were increased by 50 to 140-fold. In addition to the dynamics, chemical shifts of the majority of the peaks were also unchanged (Fig. 4a). Taken together, phase separation concentrates SLP65 without altering its dynamic structure, particularly the regions surrounding the tyrosine phosphorylation sites that bind downstream effectors.

Multivalent SH3 domain-mediated interactions have been shown to drive protein phase separation^21^. CIN85’s SH3 domains bind to multiple PRMs on SLP65 with different affinities (Fig. 4c–d). Amide resonance peaks of the first three PRMs (i.e. V47, A115, and V164) were shifted progressively in the titration series, indicating fast exchange between bound and unbound forms (Fig. 4d, left). Conversely, the peaks of the last three PRMs (i.e. S243, E279, and R312) were broadened beyond detection, which is indicative of tighter bindings (Fig. 4d, right). At 15 μM concentrations of SLP65 and CIN85_Δ57_, where phase separation has already occurred, only the high-affinity PRMs were involved in the interactions (Fig. 4c, depicted in orange and cyan). These data suggest that at low concentrations of SLP65 and CIN85, the high-affinity PRMs are the major interaction sites that drive phase separation in vivo, while the others remain accessible to different binding partners of SLP65. To investigate their binding specificity, we used paramagnetic relaxation enhancement to probe the interactions between a targeted PRM of SLP65 and the three SH3 domains of CIN85. The paramagnetic relaxation data indicate that all SH3 domains bind to the strongly interacting PRMs in a promiscuous manner (Supplementary Fig. 8), minimizing entropy loss when the droplet phase forms^21^. In summary, these data showed that the structural parameters of the interaction between SLP65 and CIN85 obey to those found for other phase-separating entities.

## Discussion

Liquid-liquid phase separation of proteins and RNA^18–20^ has been reported to underlie the formation of various functionally important nuclear and cytoplasmic membrane-less organelles^27–31^. The tripartite phase separation of B-lymphoid signaling elements described here represents a concept in BCR signal orchestration, which is unique for several reasons. First, at physiological concentrations, both proteins and vesicles are essential for phase separation. This concerted action is different from the recently described synaptic vesicles recruitment to already phase-separated Synapsin 1^32^. Second, phase separation of SLP65 and CIN85 on the surface of highly curved cytosolic vesicles differs from clustering of the transmembraneous proteins Linker for Activation of T cells (LAT)^33^ and Nephrin^34^ at the relatively flat plasma membrane. Third, SLP65 has a rather low partition coefficient in the tripartite system compared to other phase-separating proteins studied so far. The partition coefficient may depend on several factors including the type, valency, and strength of the interactions involved. Indeed, the fluidity of SLP65 is reduced only marginally in the phase-separated state to participate in dynamic signal processes. Qualitatively, the mean distance between the vesicles of 5 nm and even the most extreme distance of 20 nm measured by cryo-ET fall within the estimated lengths of two intrinsically disordered SLP65 molecules, each bound to different vesicles and held together by CIN85 molecules. Although vesicles co-phase separate together with SLP65 and CIN85 in vitro and our results demonstrate a strong correlation between the phase separation transition threshold in vitro and the granules formation in B cell, it will be important to further investigate the vesicle distribution in the granules within the B cell by in situ approaches. Nevertheless, vesicle-driven self-organization of signal transducing elements into granules has not been described before as a mode of subcellular compartmentalization.

Signal transducers often have a modular architecture where multiple interaction domains are flexibly linked and thus can integrate inputs from different proteins to tune their signals. Meanwhile, multivalency and structural disorder are also properties that can promote their phase separation, as shown here^19–21,31^. Hence, phase transition may be an additional mechanism that allows even more robust spatial and temporal control of signal transduction. The engagement of preformed signaling microclusters enables their rapid engagement by ligated cell surface receptors in contrast to recruiting signal effectors in a stepwise manner. In fact, high receptor signal sensitivity is particularly needed for extracellular ligands at low concentrations, e.g. pathogens or hormones. The organization of functionally related signaling components into distinct subcellular compartments is likely to contribute to signal specificity, a phenomenon that is still enigmatic. It is conceivable that besides SLP65 and CIN85, additional BCR-controlled effectors are recruited to the granules in a dynamic manner. Future experiments on the ultrastructure, composition and transport of the vesicular signal granules are needed to address these fundamental aspects of receptor biology.

## Methods

### Sample preparations for in vitro experiments

SLP65_1–330_, SLP65_40–330_, and CIN85_1–333_ were expressed in *Escherichia coli* strain BL21(DE3) (New England Biolabs) as fusion proteins with N-terminal His_7_-tag. The proteins were first purified by metal affinity chromatography (Protino Ni-NTA, Macherey Nagel). The His_7_-tag was removed by proteolytic cleavage with TEV protease and the proteins were finally purified by size exclusion chromatography on a HiLoad 16/60 SD 75 gel filtration column (GE Healthcare). The mutants SLP65_40–330_ (C271A), SLP65_40–330_ (C271A, S141C), SLP65_40–330_ (C271A, S236C), SLP65_40–330_ (C271A, Q302C), SLP65_40–330_ (C271A, R247A, S236C) and SLP65_40–330_ (R280A) were all generated by PCR-based mutagenesis (Quikchange II, Agilent). The DNA fragment coding for CIN85_Δ57_ (deletion of residues 369-425) was made by overlap extension PCR using the primers 5′-GCGTCTAGAGGATCCATGGTGGAGGCCATAGTG GAG-3′, 5′-GTC CTC GAG GAA TTC TCA TTT TGA TTG TAG AGC TTT CTT TAT GTC G-3′, 5′-GTG TGT GTC AGC GGA CCC ACT CTT TCA GGA GGT ATC TTT TTA ATT TCA TG-3′, and 5′-CAT GAA ATT AAA AAG ATA CCT CCT GAA AGA GTG GGT CCG CTG ACA CAC AC-3′ and cloned into a modified pET28a vector (Novagen). Protein expression and purification was performed as described above. Protein samples of different SLP65 and CIN85 constructs were finally transferred by dialysis into buffer containing 20 mM HEPES, pH 7.2, 100 mM NaCl, 1 mM TCEP, and 0.5 mM Pefabloc.

Atto 430LS dye (Atto-Tec) was conjugated to either SLP65_1-330_ or SLP65_40–330_ at residue C271 via maleimide thiol reaction. After 2-h incubation, unbound dye was removed by size exclusion chromatography (Superdex 75 10/300 GL). The final protein concentration was determined by UV absorbance at 280 nm, taking into account the dye’s maximum absorbance at 430 nm and its relative absorbance at 280 nm.

To prepare the vesicles, a total molar concentration of 5 mM phospholipids with the different phospholipid compositions (Avanti Polar Lipids) was mixed in chloroform, dried under nitrogen stream, and lyophilized overnight. The lipid film was then resuspended in the HEPES buffer, sonicated extensively, and extruded through 50-nm and 100-nm filters (Avanti Polar Lipids) to make 20-nm SUVs and 60-nm LUVs, respectively. The vesicle solution was diluted to a final phospholipids concentration of 1 mM for the in vitro reconstitution experiments. The hydrodynamic radius was determined by dynamic light scattering (Wyatt Technology).

### Confocal fluorescence microscopy

Atto 430LS-tagged SLP65_1–330_, CIN85_1–333_ or CIN85_Δ57_, and SUVs were mixed at the indicated concentrations in a 20-μl volume, incubated at room temperature for over 1 h, and then transferred to uncoated 8-well μ-Slide (ibidi) for microscopy. Confocal fluorescence and bright-field microscopy were performed using Leica TCS SP5 microscope with a water immersion objective (Leica HCX PL APO CS 63 × /1.20). Atto 430LS-tagged SLP65_1–330_ was illuminated by 458-nm laser (5 to 20% transmitted power) and observed within the bandwidth of 500–600 nm. Bright-field imaging was performed using 2% laser power of 488 nm and observed via a bright-field filter. The images were acquired at 400 Hz without line averaging. For live-cell imaging, cells were resuspended in serum-free buffer and seeded in glass-covered dishes. Images were recorded using a Leica TCS SP2 LSM. Fluorescence images were analyzed using ImageJ. The single side dimension of each region of interest (ROI) was either 61.63 μm (for SLP65’s concentration ≥ 5 μM) or 20.54 μm (for SLP65’s concentration <2 μM). The pixel size was either 481.5 nm or 160.5 nm, respectively. To quantify fluorescence intensities inside and outside of the droplets, first the “Threshold” function was applied using default algorithm, and then a mask for the droplets was generated from the binary image using the “Analyze Particles” function. The “Size” parameter had to be adjusted to include all visually distinguishable droplets and the “Circularity” parameter was set as 0.1–1.0. Index of dispersion (i.e. ratio of intensity variance to mean) and partition coefficient (i.e. ratio of intensity inside to outside of droplets) were calculated by Matlab scripts.

### Cells and expression vectors and antibodies

DG75 B cells (DSMZ ACC83) were cultured in RPMI 1640 supplemented with 10% fetal calf serum, 3 mM L-glutamine, 1 mM sodium pyruvate, 50 µM 2-mercaptoethanol and antibiotics. DT40 cells (ATCC CRL-2111) were cultured in medium containing 1% chicken serum and no pyruvate. BCR stimulation of the cells was performed with 0.5 µg/ml of F(ab’)_2_-anti-human IgM (Jackson ImmunoResearch) at 37 °C or 2 µg/ml anti-chicken-IgM (clone M4; Southern Biotechnology) at room temperature.

The cDNAs encoding human CIN85 and SLP65, respectively, were cloned into the pMSCV vector (BD Biosciences) containing the sequence for the EGFP variant citrine, resulting in N-terminal citrine-tagged proteins. In addition to the wild-type protein-encoding constructs, similar cloning was performed to express variants. The CIN85-Δ57 variant lacks amino acid residues 369–425, CIN85-ΔCC the amino acid residues 594–665, SLP65_ΔN_ amino acids 1–45, and SLP65_ΔPRM_ harbors R-to-A exchanges as described previously^4^. To express the N-BAR-SLP65_ΔN_ chimeric protein, construct encoding the amino acid residues 2–239 of murine Amphiphysin N-terminally fused to SLP65_ΔN_ was generated by overlap extension PCR using the primers 5′-TAATAGATCTCGCCGACATCAAGACGGGCATCT-3′, 5′-GCTCTCTGAAGCGTAGTCGTCAGCATGCTGATCACCGAGT-3′, 5′-GAGCAGTGGTGACTGGTGACTACGCTTCAGAGAGCCCTG-3′, and 5′-TAATGCGGCCGCCTACTCCAGGCGCCGCGTGAA-3′. Empty EGFP encoding vector served as the control. The resulting expression plasmids were transduced into the CIN85-deficient DG75 B cells and SLP65-deficient DT40 cells, respectively. Transduced cells were subsequently subjected to Western Blot, Ca^2+^ flux, or imaging analyses. Utilized antibodies were diluted 1:1000 and are specific for SLP65 (clone 2B11, Santa Cruz Biotechnology sc-8003), CIN85 (clone D1A4, Cell Signalling Technology 12304), CD2AP (R&D Systems AF4474), or actin (clone 13E5, Cell Signalling Technology 4970). Cytosolic Ca^2+^ concentrations were monitored by flow cytometric analysis of cells that were loaded with 1 µM Indo1-AM (Thermo)^3^.

### Cryo-electron tomography

*Sample preparation*: for cryo-ET experiments, proteins (1 μM SLP65_1–330_ and 1 μM CIN85_Δ57_) and SUVs (1 mM) were mixed in an Eppendorf tube and incubated for 45 min. In all, 4 µL of said suspension were carefully applied to R2/1 copper TEM grids (Quantifoil), which were subsequently plunge frozen on a Vitrobot Mark IV (FEI, settings: blot force = 10, blot time = 10 s, temperature = 21 °C, humidity = 90%). The samples were stored under LN_2_ until use. Vesicle diameters are not changed by the preparation procedure^35^.

*Data acquisition*: the grids were screened for sites suitable for cryo-ET in low magnification and areas were chosen that still allowed for data acquisition at reasonable exposure timings (thickness <200–300 nm). Cryo tomograms were obtained on a transmission electron microscope (Titan Krios, FEG 300 kV, FEI) with a post-column energy-filter (968 Quantum K2, Gatan) using a defocus range of −5 to −3.5 µm and an EFTEM magnification of ×42,000 (calibrated pixel size 3.42 Å). Images were recorded with a direct electron detector camera (K2 Summit, Gatan) in dose-fractionation mode and a total dose of ~80 e^−^/Å^2^ per tomogram using SerialEM^36^. The acquisition was controlled by an in-house script using a dose-symmetric tilt scheme^37^ with an angle increment of 2°. The stage was tilted between 60° and −60° starting at 0°. Frames were aligned using MotionCor2^38^, and tomogram reconstruction was performed in IMOD^39^.

*Visualization*: to enhanced contrast of the TEM insets, summed projections of five slices each are shown. Vesicles were manually segmented for 3D-visualization in AMIRA 6.2 (ThermoFisher) and processed using an in-house python script and Matlab (Mathworks).

### NMR spectroscopy

^15^N-labeled SLP65_1-330_ and CIN85_1-333_ were expressed in M9 minimal medium supplemented with ^15^N-NH_4_Cl as nitrogen source. Protein expression and purification was performed as described above. The samples were finally transferred by dialysis into buffer containing 20 mM HEPES, pH 7.2, 100 mM NaCl, 1 mM TCEP, and 0.5 mM Pefabloc. In total, 4% D_2_O and 0.5 mM DSS were added as internal reference and the samples were kept inside 3-mm Shigemi or normal NMR tubes.

For PRE measurements, either SLP65_40-330_ that naturally contains a single cysteine (C271), or other individual cysteine mutants on the SLP65_40-330_ (C271A) construct (see above) were ligated with the nitroxide spin label MTSL (1-oxy-2,2,5,5-tetramethyl-d-pyrroline-3-methyl)-methanethiosulfonate (Toronto Research Chemicals, Toronto)^40^.

All NMR measurements were performed at 293 K on Bruker’s 800 MHz and 900 MHz spectrometer that are equipped with a z-gradient cryoprobe. The sensitivity-enhanced ^15^N-HSQC with water-flipback pulses were measured with a recycle delay of 1 s and indirect-dimension *t*_max_ of 55–70 ms (NS = 8–140, cosine-squared function in both dimensions). The residue-specific ^15^N transverse cross-correlated relaxation rates were measured using 2D TRACT experiment^15,41^ with variable delays (NS = 8–80, cosine-squared function in both dimensions) and fitted to single exponential decay. The ^1^H *R*_2_-PRE was measured on ^15^N-labeled CIN85_1-333_ mixed with MTSL-tagged SLP65 constructs at 10:1 molar ratio using a two-point delay (Δ*t* = 7 ms) in the first INEPT period of a TROSY experiment^42^ (NS = 64–80, cosine-squared function in both dimensions) and analyzing the peak intensities. The corresponding SLP65 constructs without MTSL were used as diamagnetic reference in the presence of 1 mM TCEP.

## Supplementary information

supplementary information

## Data Availability

Data supporting the findings of this manuscript are available from the corresponding authors upon reasonable request. A reporting summary for this Article is available as a Supplementary Information file. The source data underlying Figs. 1c–e, 2b, 3b, c, 4c, and Supplementary Figs. 1, 2, 3, 4, 6b, d, and 7 are provided as a Source Data file.

## Source data

Source Data
