# Supplementary material for: Tripartite phase separation of two signal effectors with vesicles priming B cell responsiveness
Source: Nat Commun. 2020 Feb 12;11:848. doi: 10.1038/s41467-020-14544-1 (PMC7016142; doi:10.1038/s41467-020-14544-1)
Supplement: Supplementary file 1 — supplementary information [file 41467_2020_14544_MOESM1_ESM.pdf]

Supplementary Information for manuscript

Tripartite phase separation of two signal effectors with vesicles priming B cell responsiveness

By Wong *et al.*

Supplementary Figures

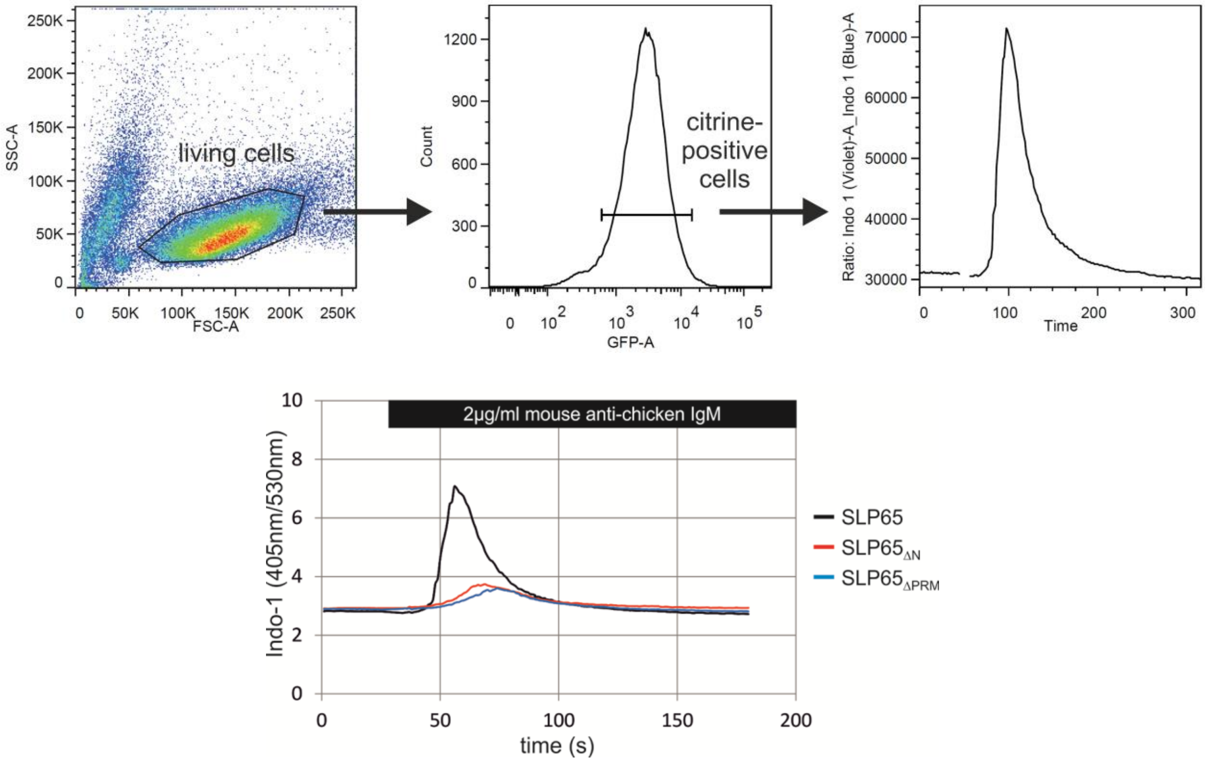

Supplementary Figure 1. SLP65 variants that abrogate the BCR-induced calcium flux response. SLP65-deficient chicken DT40 B cells, which were reconstituted with different SLP65 variants as described in the main text (Fig. 1a), were loaded with the Ca^{2+}-sensitive fluorophore Indo-1 and subjected to flow cytometric analysis. The general gating strategy is depicted in the upper part of the figure. First, the living cell population was gated according to the FCS/SSC pattern (left image), then the citrine-positive cells were selected on the basis of their fluorescence signal in the GFP channel (middle image), and subjected to kinetic analyses (right image). Cells were stimulated with 2 µg/ml anti-chicken IgM (M4) at the indicated time and the relative Ca^{2+} concentration is monitored via the ratio of fluorescence intensities at 405 nm and 530 nm. Source data are provided as a Source Data file.

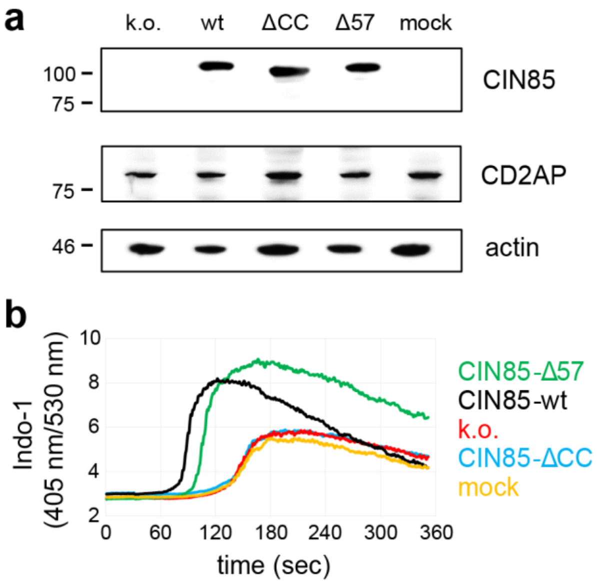

Supplementary Figure 2. CIN85$_{\Delta57}$ can functionally substitute wild-type CIN85 in human DG75 B cells. (a) Immunoblot analyses with antibodies to CIN85, CD2AP or actin of surface IgM-expressing DG75 cells that were rendered deficient for the expression of CIN85 (k.o.) by CRISPR/Cas9 genome editing or cells that were subsequently transduced with constructs encoding citrine-tagged versions of wild-type CIN85 (wt), or CIN85 deletion mutants lacking either amino acid residues 369 to 425 (Δ57) or the C-terminal coiled coil domain (ΔCC). Transduction with EGFP alone served as additional control (mock). Relative molecular masses of marker proteins are indicated on the left in kDa. (b) Flow cytometric Ca^{2+} mobilization profiles of these cells were recorded upon BCR ligation with 0.5 μg/ml anti-IgM F(ab')2 fragments (n=3). Source data are provided as a Source Data file.

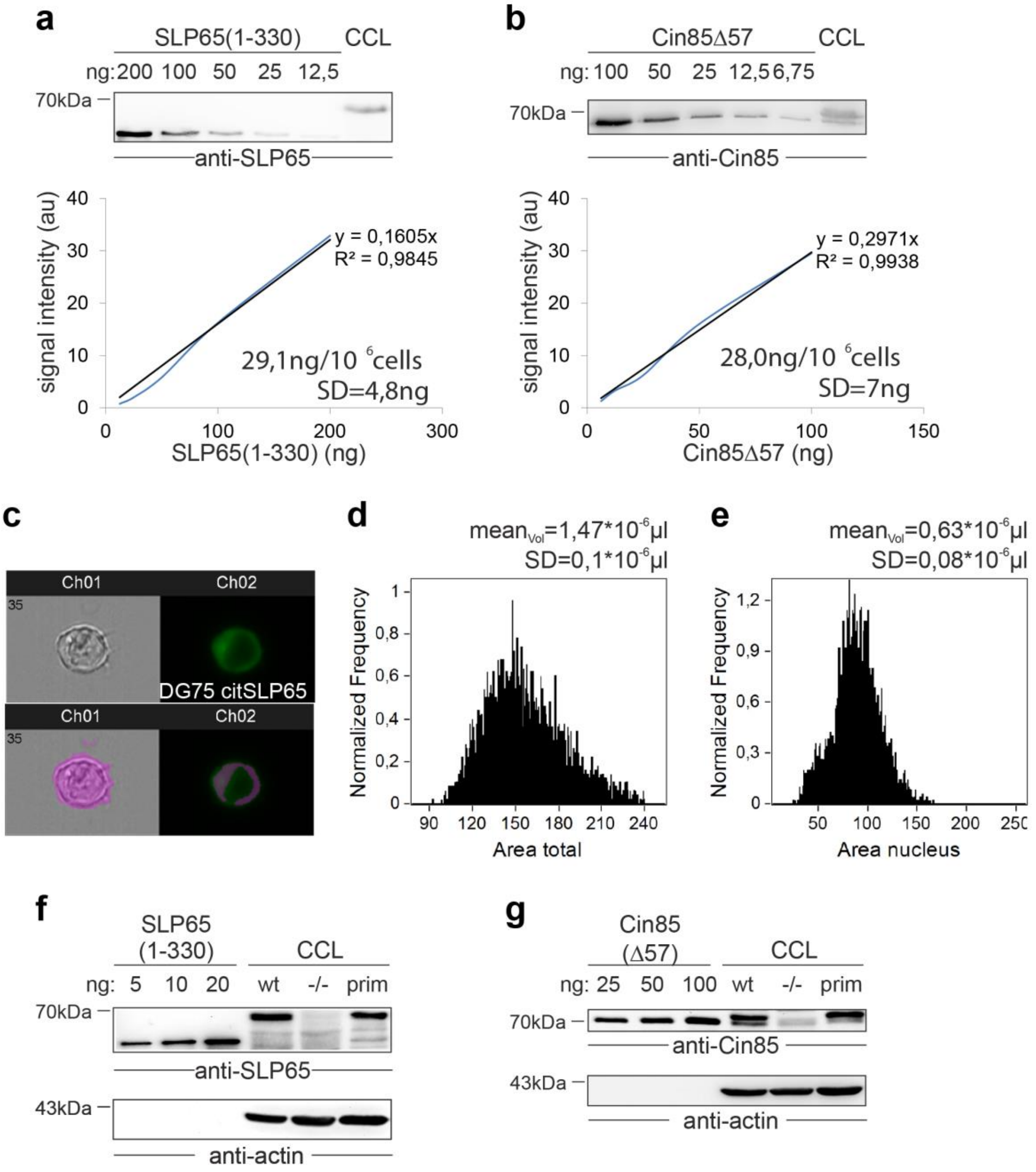

Supplementary Figure 3. Quantification of the molarity of SLP65 and CIN85 in DG75 and primary human B cells. The amount of SLP65 (a) or CIN85 (b) in wild-type DG75 cells was determined by Western Blot analysis using antibodies to SLP65 (2B11, SCBT) or CIN85 (D1A4, Cell Signaling Technology), respectively. Recombinant proteins comprising either the first 330 residues of SLP65 ($SLP65_{1\text{-}330}$) or the CIN85 construct with a 57-amino acid deletion starting at residue 369 ($CIN85_{\Delta 57}$) were subjected to SDS-PAGE at the indicated amounts together with cleared cellular lysates (CCL) from 10^6 DG75 cells. After development of the Western Blots, the signal intensities were quantified (ImageJ) and linear regression was performed as exemplified in the lower parts of (a) and (b). Each regression line's slope was calculated together with the coefficient of correlation to determine the amounts of SLP65 and CIN85, respectively, as indicated in the diagrams. Values and standard deviations (SD) were calculated from n≥5 independent experiments. (c) To determine the volume of the cytosol in

DG75 cells, imaging flow cytometry was applied (Amnis ImageStream X100, Millipore) to DG75 cells expressing a citrine-tagged version of SLP65 as a marker for the cytosol. Wide field and fluorescent images were taken as exemplified in the upper panel of (c). For analysis (Ideas software, Amnis) masks were generated covering either the whole cell (lower panel, ch01) or the cytosolic area based on the SLP65 fluorescence (lower panel, ch02). Based on the masks depicted in (c), the mean values for the total area of cells (d) and the mean area of the nucleus (e) were determined from n=5000 cells that were selected for maximal sharpness, allowing for the calculation of the respective volumes indicated in the histograms ($mean_{Vol}$ ± SD), which correspond to a cytosolic $mean_{Vol} = 0.84*10^{-6}\ \mu l \pm 0.17*10^{-6}\ \mu l$. Based on these values together with the endogenous protein amounts determined in (a) and (b), the molarity of SLP65 (530 nM ± 90 nM) and CIN85 (470 nM ± 190 nM) was calculated, respectively. To verify similar expression of SLP65 and CIN85 in DG75 cells and primary human B cells, we isolated B cells from peripheral blood mononuclear cells using the MojoSort™ Mouse Pan B Cell kit (Bio Legend) and prepared CCLs from $2*10^6$ cells. Indicated amounts of recombinant proteins used in (a) and CCLs from wild-type and SLP65- (f) or CIN85 (g)-deficient DG75 cells served as positive and negative controls, respectively. Samples were subjected to Western Blot analyses using antibodies against SLP65 (f, upper panel) and CIN85 (g, upper panel). The blots were reprobed with anti-actin antibodies to confirm similar loading. Source data are provided as a Source Data file.

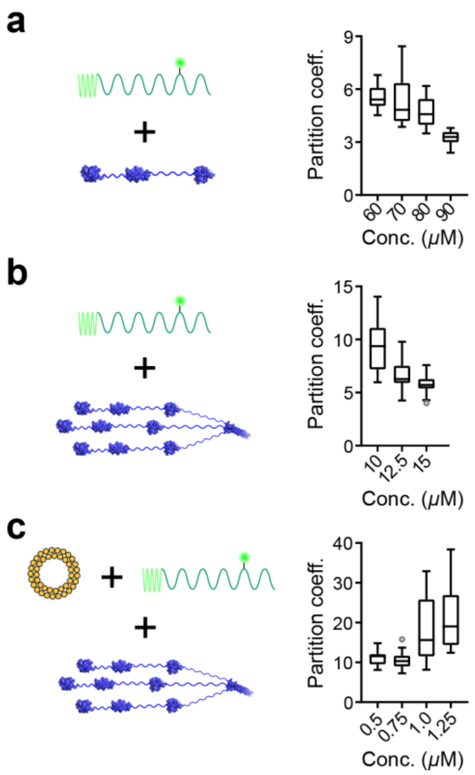

Supplementary Figure 4. Partition coefficients of phase-separated SLP65. The partition coefficients shown as box-and-whisker plots (12 ROIs and 2 independent samples, n = 24) were determined from the fluorescence intensities inside and outside of the droplets measured on a mixture of different concentrations of Atto 430LS-tagged SLP65$_{1\text{-}330}$ with the corresponding equal concentrations of either (a) CIN85$_{1\text{-}333}$, (b) CIN85$_{\Delta 57}$, or (c) CIN85$_{\Delta 57}$ and 1 mM of SUVs. Concentrations of CIN85$_{\Delta 57}$ refer to the monomeric concentration. The line inside the box and the edges of the box correspond to the median, first and third quartiles, respectively. The outliers with value $\geqslant$ 1.5 times the interquartile range away from the top or bottom of the box are denoted by circles. Source data are provided as a Source Data file.

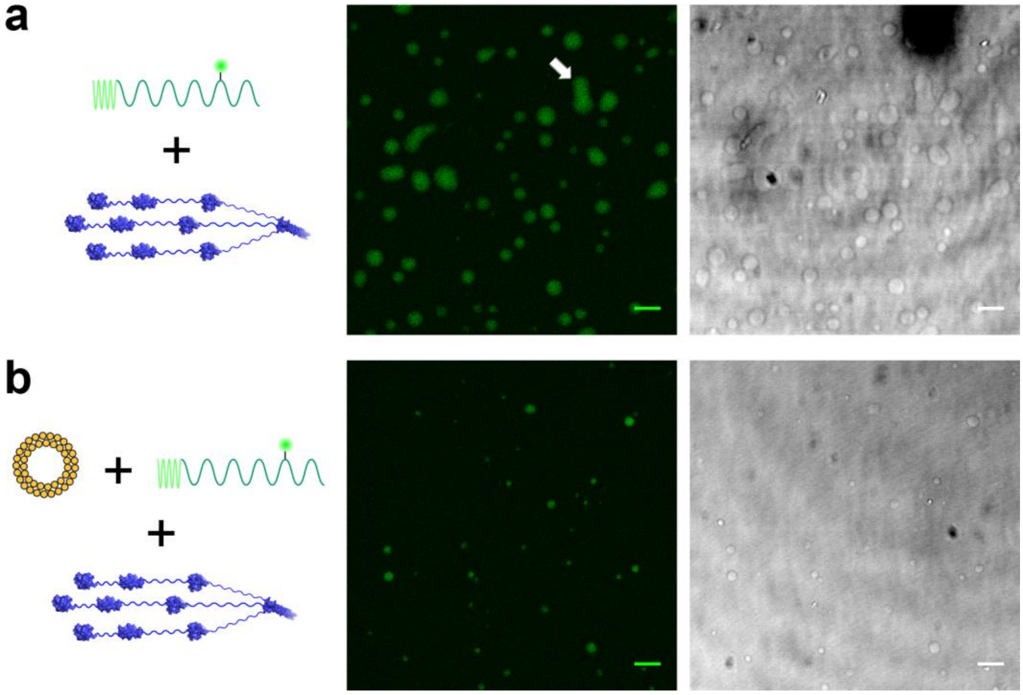

Supplementary Figure 5. Microscopy of the droplets. Confocal fluorescence (*left*) and bright-field (*right*) microscopy images of a mixture of (a) 15 μM Atto 430LS-tagged SLP65$_{1-330}$ and 15 μM CIN85$_{\Delta 57}$, and (b) 1.5 μM Atto 430LS-tagged SLP65$_{1-330}$ and 1.5 μM CIN85$_{\Delta 57}$ together with 1 mM of SUVs. Droplets undergoing fusion are pointed by a white arrow in (a). (Scale bar = 10 μm)

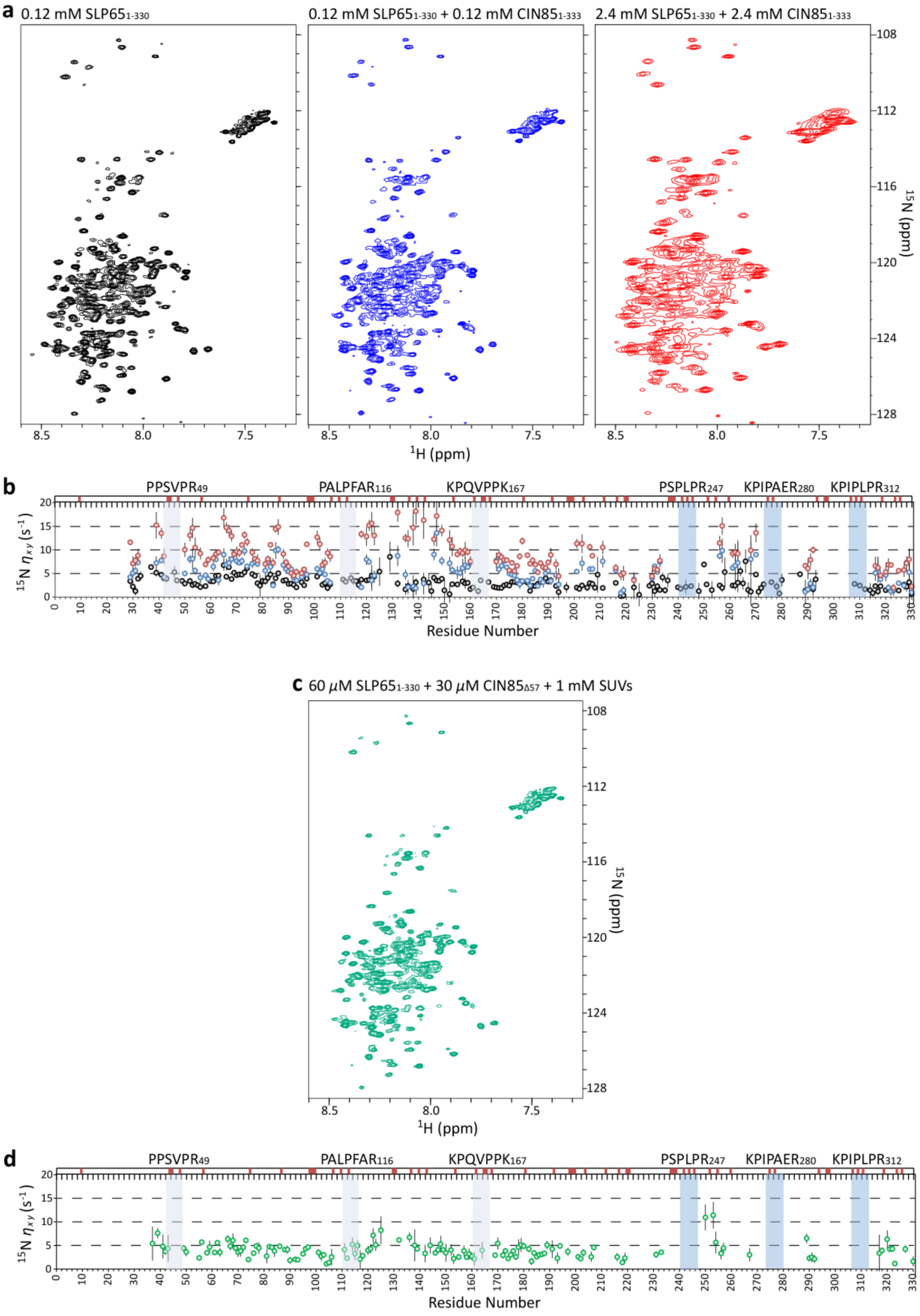

Supplementary Figure 6. ^{15}N relaxation rates of SLP65 mixed with CIN85 at different concentrations. (a) ^{15}N-HSQC spectra of 0.12 mM ^{15}N-labeled SLP65 only (black), the

mixture of 0.12 mM (blue) or 2.4 mM (red) of ^{15}N-labeled SLP65 and unlabeled CIN85$_{1\text{-}333}$ at equal concentrations, respectively. (b) Residue-specific ^{15}N transverse cross-correlated relaxation rate (^{15}N η_{xy}), which is independent of the chemical exchange effects, was measured using the 2D TRACT experiment on the samples described in (a). Error bar indicates standard error of the fitted parameter. The average relaxation rates were increased from 3.0 s^{-1} (black) to 5.5 s^{-1} (blue) to 9.3 s^{-1} (red). Proline residues were marked in red on top of the chart and PRMs that interact with CIN85 were highlighted as in the main text Figure 4. Source data are provided as a Source Data file. (c) ^{15}N-HSQC spectrum of the mixture of 60 μM ^{15}N-labeled SLP65, 30 μM unlabeled CIN85$_{\Delta 57}$ and 1 mM SUVs. (d) Residue-specific ^{15}N η_{xy} was measured on sample (c) and the average relaxation rate was 3.9 s^{-1}. Error bar indicates standard error of the fitted parameter. Source data are provided as a Source Data file.

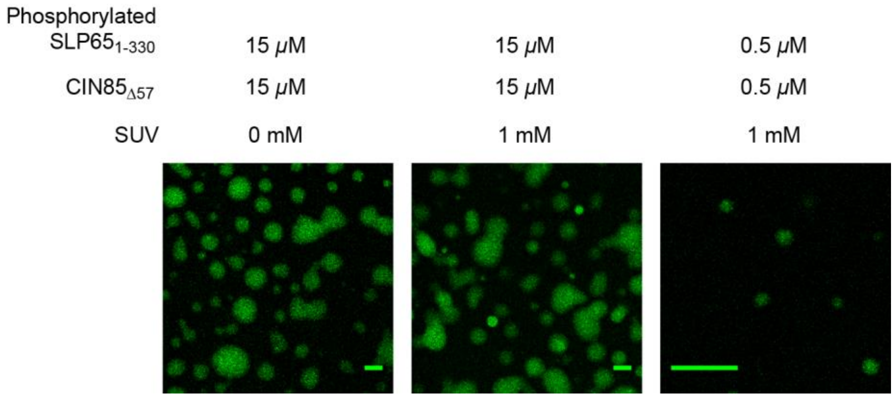

Supplementary Figure 7. Phase separation of phosphorylated SLP65. Confocal fluorescence microscopy of a mixture of Atto 430LS-tagged Syk-phosphorylated SLP65$_{1\text{-}330}$, CIN85$_{\Delta 57}$, and SUVs (DOPC:DOPE:DOPS = 65:25:10 mol%) with the corresponding concentration of each component indicated on top of the images. Phosphorylation of the five tyrosine residues by Syk was monitored by NMR spectroscopy in a separate sample. (Scale bar = 10 μm) Source data are provided as a Source Data file.

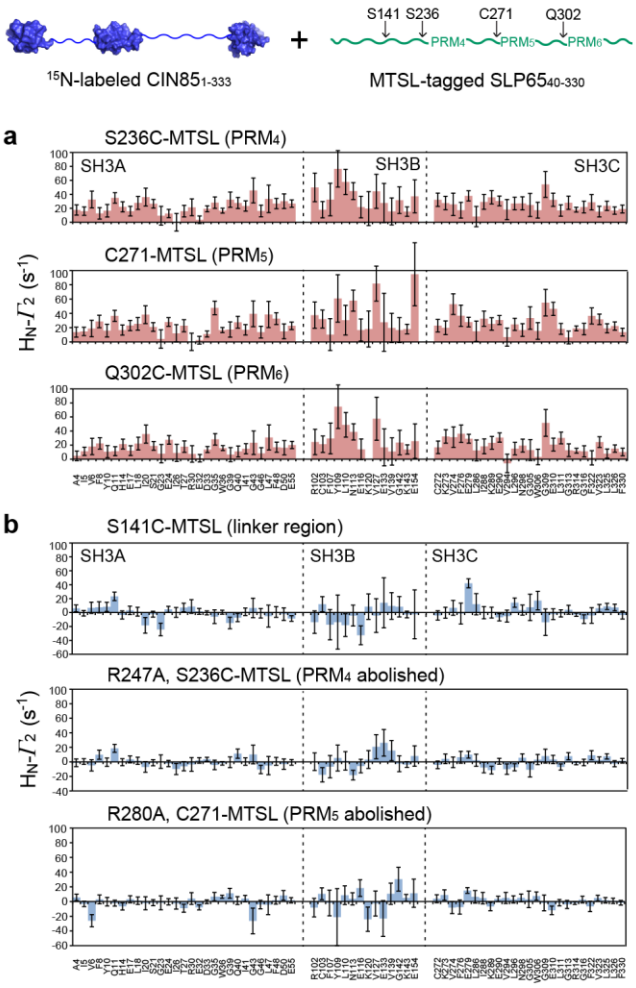

Supplementary Figure 8. Promiscuous interactions between CIN85's SH3 domains and SLP65's PRMs. (a) Intermolecular paramagnetic relaxation enhancement (PRE) of the amide proton's R_2 of the three SH3 domains of CIN85$_{1\text{-}333}$ was induced by unlabeled MTSL-tagged SLP65$_{40\text{-}330}$, where MTSL was positioned close to either one of the high-affinity binding PRMs, namely PRM$_4$, PRM$_5$, and PRM$_6$. (b) PRE measurements were also performed on the sample where MTSL was positioned at S141 away from any of the PRMs. In addition, MTSL was attached close to PRM$_4$ or PRM$_5$ that contain single mutation R247A or R280A, and thus do not bind to the SH3 domains. Error bar indicates uncertainty propagated from the peak's signal-to-noise.
